# IL-17 inhibits CXCL9/10-mediated recruitment of CD8^+^ cytotoxic T cells and regulatory T cells to colorectal tumors

**DOI:** 10.1186/s40425-019-0757-z

**Published:** 2019-11-27

**Authors:** Ju Chen, Xiaoyang Ye, Elise Pitmon, Mengqian Lu, Jun Wan, Evan R. Jellison, Adam J. Adler, Anthony T. Vella, Kepeng Wang

**Affiliations:** 1 Department of Immunology, School of Medicine, UConn Health, 263 Farmington Ave, Farmington, CT 06030 USA; 2 Shenzhen Key Laboratory for Neuronal Structural Biology, Biomedical Research Institute, Shenzhen Peking University - The Hong Kong University of Science and Technology Medical Center, Shenzhen, China; 3 School of Acupuncture-moxibustion and Tuina, Beijing University of Chinese Medicine, Beijing, China; 4 Division of Life Science, The Hong Kong University of Science and Technology, Clear Water Bay, Hong Kong, China

**Keywords:** Interleukin-17, CXCL9, CXCL10, Regulatory T cell, And colorectal cancer

## Abstract

**Background:**

The IL-17 family cytokines are potent drivers of colorectal cancer (CRC) development. We and others have shown that IL-17 mainly signals to tumor cells to promote CRC, but the underlying mechanism remains unclear. IL-17 also dampens Th1-armed anti-tumor immunity, in part by attracting myeloid cells to tumor. Whether IL-17 controls the activity of adaptive immune cells in a more direct manner, however, is unknown.

**Methods:**

Using mouse models of sporadic or inducible colorectal cancers, we ablated IL-17RA in the whole body or specifically in colorectal tumor cells. We also performed adoptive bone marrow reconstitution to knockout CXCR3 in hematopoietic cells. Histological and immunological experimental methods were used to reveal the link among IL-17, chemokine production, and CRC development.

**Results:**

Loss of IL-17 signaling in mouse CRC resulted in marked increase in the recruitment of CD8^+^ cytotoxic T lymphocytes (CTLs) and regulatory T cells (Tregs), starting from early stage CRC lesions. This is accompanied by the increased expression of anti-inflammatory cytokines IL-10 and TGF-β. IL-17 signaling also inhibits the production of T cell attracting chemokines CXCL9 and CXCL10 by tumor cells. Conversely, the inability of hematopoietic cells to respond to CXCL9/10 resulted in decreased tumor infiltration by CTLs and Tregs, decreased levels of IL-10 and TGF-β, and increased numbers of tumor lesions. Blockade of IL-17 signaling resulted in increased expression of immune checkpoint markers. On the other hand, treatment of mouse CRC with anti-CTLA-4 antibody led to increased expression of pro-tumor IL-17.

**Conclusion:**

IL-17 signals to colorectal tumor cells and inhibits their production of CXCL9/10 chemokines. By doing so, IL-17 inhibits the infiltration of CD8^+^ CTLs and Tregs to CRC, thus promoting CRC development. Cancer immunotherapy may be benefited by the use of anti-IL-17 agents as adjuvant therapies, which serve to block both IL-17-mediated tumor promotion and T cell exclusion.

## Background

The IL-17 family cytokines promote tumor development in multiple organs. Using mouse models of sporadic and inducible colorectal cancers (CRC), we and others have shown that IL-17 signals to transformed colorectal epithelial cells to drive tumor development [1, 2]. This IL-17-tumor cell signaling is necessary for the survival and outgrowth of early CRC lesions, and ablation of IL-17RA, the common receptor of IL-17 family cytokines, resulted in marked reduction in tumor numbers in mouse colon [1, 3]. IL-17 also activates production of chemokines, such as CXCL1 and CXCL2 that attract myeloid cells to sites of inflammation [4, 5]. Colorectal tumor cells exhibit defective epithelial barrier function. As a result, gut commensal bacteria and their degradative products invade tumor stroma, engage tumor-infiltrating myeloid cells, and activate the production of IL-23 and its downstream cytokine IL-17 [3]. Thus, the IL-17-myeloid cell pathway forms a self-enhancing loop that results in chronic tumor-associated inflammation. IL-17 is also known to block the effect of cytotoxic and anti-angiogenic chemotherapies against colorectal cancers [1, 6]. This correlates with the observation that loss of IL-17 signaling resulted in enhanced recruitment of CD8^+^ cytotoxic T lymphocytes (CTL) [1, 3, 7]. To date, it is unclear if IL-17 regulates the recruitment of adaptive immune cells to the site of CRC, and if so, what the underlying mechanism is.

The chemokine CXCL9 signals through CXCR3 and mediates migration of T cells to sites of inflammation [8]. In mouse models of transplanted tumors, CXCR3 signaling promotes CD8^+^ T cell infiltration that controls tumor growth [9–11]. The role of CXCL9 and its family members in sporadic CRC is unknown. Chemokine signaling through CXCR3 also mediates the recruitment of CD4^+^ T cells. Among them, Th17 cells promote CRC development by secreting IL-17 and IL-22 [1, 3, 12, 13], while Th1 cells have long known to inhibit tumor development [14]. Perhaps most intriguingly, regulatory T cells (Tregs) inhibit CRC development by dampening tumor-promoting inflammation [15]. Ablation of Treg-related cytokines IL-10 and TGF-β resulted in increased intestinal tumor burden [16, 17]. A high “Treg signature” in human CRC also indicates better prognosis [18]. The function of the CXCR3 cascade in CRC thus depends on the immune cell types that they recruit. The unique Treg-CRC relationship also complicates the use of Treg-targeting strategies, such as anti-CTLA4 for CRC treatment [19].

Here we show that IL-17 signals to transformed epithelial (tumor) cells to suppress the expression of CXCL9 and CXCL10 chemokines. Signaling of CXCL9/10 through CXCR3 is required for the recruitment of CD8^+^ CTLs and Tregs, but not Th1 or Th17 cells, to colorectal tumors. CXCR3 signaling to hematopoietic cells is required for the expression of IL-10 and TGF-β in tumors, and for the control of CRC development. Overall, IL-17 promotes CRC development by suppressing cells responsible for anti-cancer immunity, and fostering tumor-promoting gut inflammation. This novel mechanism pinpoints gut inflammation during cancer as a barrier for tumor control through the diverting action of IL-17 on the adaptive immune system.

## Methods

### Animal models

*Il17ra*^−/−^ mice were from Amgen [20]. C57BL/6, *Apc*^*F/F*^ [21], *Cd8a*^−/−^ [22], *B2m*^−/−^ [23], *Cdx2-Cre* [24], *Cdx2-Cre-ERT2* [25], and *Cxcr3*^−/−^ [26] mice were obtained from the Jackson Laboratory. *Il17ra*^F/F^ mice [1] were obtained from Dr. Michael Karin’s laboratory at University of California, San Diego.

To generate the mouse model of sporadic CRC, *Cdx2-Cre* and *Apc*^*F/F*^ mice were crossed to generate *Cdx2-Cre*^*+*^/ *Apc*^*F/WT*^ mice. These mice were sacrificed around 5 months of age for tumor analyses. Mouse colon was dissected, and colorectal tumors were excised with a scissor. Tumor-adjacent colon tissues were harvested and analyzed as “normal colon tissue” for comparison.

For tamoxifen-inducible tumorigenesis, *Cdx2-Cre-ERT2*^*+*^*/Apc*^*F/F*^ mice were given 75 mg/kg body weight tamoxifen (Sigma, dissolved in 5% ethanol, 95% corn oil) *i.p*. on a daily basis for 3 consecutive days. Mice were sacrificed 4 to 5 weeks after the last dose of tamoxifen for tumor statistics and analysis. Mouse colon was dissected, and visible colorectal tumors (typically 1–2 mm in diameter) were excised with a scissor.

All mice were maintained in filter-topped cages on autoclaved food and water at UConn Health. All experiments used co-housed, gender matched littermates to ensure consistency of common microflora. Both male and female mice were used for all experiments.

### Bone marrow transplantation

Six- to eight-week-old recipient mice were irradiated twice during 1 day to achieve a lethal dose (2 × 600 rad) and intravenously injected with single-cell suspension of 10^7^ donor bone marrow cells. Recipients were co-housed littermates, which were transplanted with both gene-deficient and wild-type bone marrow for comparison. After transplantation the recipients were placed on sulphamethoxazole and trimethoprime in drinking water for 2 weeks, followed by regular water. Mice were sacrificed and analyzed for tumor development 4–5 months after transplantation.

### Antibody treatment in mice

For sporadic CRC model (*Cdx2-Cre*^+^/*Apc*^F/WT^ mice), IL-17A, CTLA-4, and PD-1 neutralizing antibodies or isotype control antibodies (Bio X Cell) were *i.p.* injected at a dose of 100 μg per mouse every 3 days until sacrifice.

For the tamoxifen inducible model of tumorigenesis, antibodies (100 μg per mouse, every 3 days) were injected 1 day after the dose of tamoxifen until sacrifice.

### Immunofluorescent staining and ELISA

Immunofluorescent staining was performed on cryosectioned colorectal tumors with antibody against CD8α (Thermo Fisher), followed with Alexa-488-conjugated secondary antibody (Life Technology). Sections were further stained with DAPI and imaged under a confocal microscopy. For ELISA analysis of CXCL9 (Biolegend) and CXCL10 (R&D Systems), colonic tumors were cultured in opti-MEM containing 1% Antibiotic-Antimycotic (Life Technologies) for 24 h. Tissue culture supernatant was analyzed by ELISA. Concentrations of chemokines were normalized to the weight of tumors in each well.

### Cell culture and cytokine treatment

Primary CRC tumor sphere culture was previously described [1]. Briefly, tumor cells were isolated from colorectal tumors of *Cdx2-Cre-ERT2*^+^*/Apc*^F/F^ mice 4 weeks after tamoxifen injection. Cells were plated in Matrigel (BD Bioscience) and maintained in DMEM/F12 media (Life Technologies) containing B27 and N2 supplements (Life Technologies), 1.25 mM N-acetyl L-cysteine (Sigma), 100 ng/ml noggin (Peprotech), 50 ng/ml mEGF (Biosource), and 10% Rspo1-Fc-conditioned medium. To study IL-17 signaling in vitro, tumor spheres were replenished with serum and growth factor free medium for 24 h, and treated with 100 ng/ml recombinant human IL-17A, C or F for another 24 h.

### Flow cytometry and cell sorting

Colorectal tumors were minced with scissors and digested with 1 mg/kg collagenase IV (Sigma Aldrich) for 20 min. Cells were filtered with 70-μm cell sieve, and stained with Live/Dead fixable exclusion dye (Tonbo Bioscience), followed by fluorochrome-conjugated antibodies in PBS with 2% fetal bovine serum (FBS) and 1 mM EDTA. Anti-CD3 (Cat # 100206), anti-CD4 (Cat # 100536), anti-CD45 (Cat # 103138), anti-CD19 (Cat # 152408), anti-CD11b (Cat # 101224), anti-F4/80 (Cat # 123108), anti-Gr-1 (Cat # 108428), anti-Ly6C (Cat # 128016), anti-Ly6G (Cat # 127641), anti-PD-1 (Cat # 135216), anti-Ep-CAM (Cat # 118216), anti-IL-10 (Cat # 505008), anti-IL-17A (Cat # 506904), anti-IFNγ (Cat # 505806), and anti-TNF-α (Cat # 506306) antibodies were from Biolegend. Anti-CD44 (Cat # 12–0441-82), anti-CD62L (Cat # 47–0629-42), anti-Foxp3 (Cat # 11–5773-82), and anti-Ki-67 (Cat # 11–5698-82) antibodies were from eBioscience. Anti-CD25 (Cat # 20–0251) and anti-CD3 (Cat # 20–0032) antibodies were from Tonbo Biosciences. Anti-CD8α antibody (Cat # 558106) was from BD Bioscience. For intracellular cytokine staining, cells were stimulated with Cell Stimulation Cocktail (eBioscience) for 4 h, followed with fixation and staining with Foxp3/transcription factor staining buffer set (eBioscience). Flow cytometry analyses were performed on a BD LSRII flow cytometer. Cell sorting was performed on a BD FACS ARIA II high speed cell sorter. Data was analyzed with FlowJo software.

### Q-RT–PCR analysis

Total RNA was extracted with RNeasy Plus kit (Qiagen) and reverse transcribed using an IScript kit (Biorad). Q-RT-PCR was performed using SsoAdvanced Universal SYBR Green Supermix (Biorad) on a Biorad CFX96 machine. Expression data were normalized to RPL32 mRNA levels. The data were calculated as 2^(Ct(RPL32–gene of interest))^ to compare experimental groups to controls, and are presented in arbitrary units. Primer sequences are listed in Additional file 1: Table S1. Whenever possible, primers were intron-spanning, such that amplification is feasible on complementary DNA.

### Statistical analysis

Data are presented as averages +/− S.E.M. and were analyzed by the Students’ *t* test. *P* values less than 0.05 were considered significant.

## Results

### IL-17 inhibits the infiltration of tumor-associated CD4^+^ T cells and the production of IL-10 and TGF-β

**Fig. 1:**
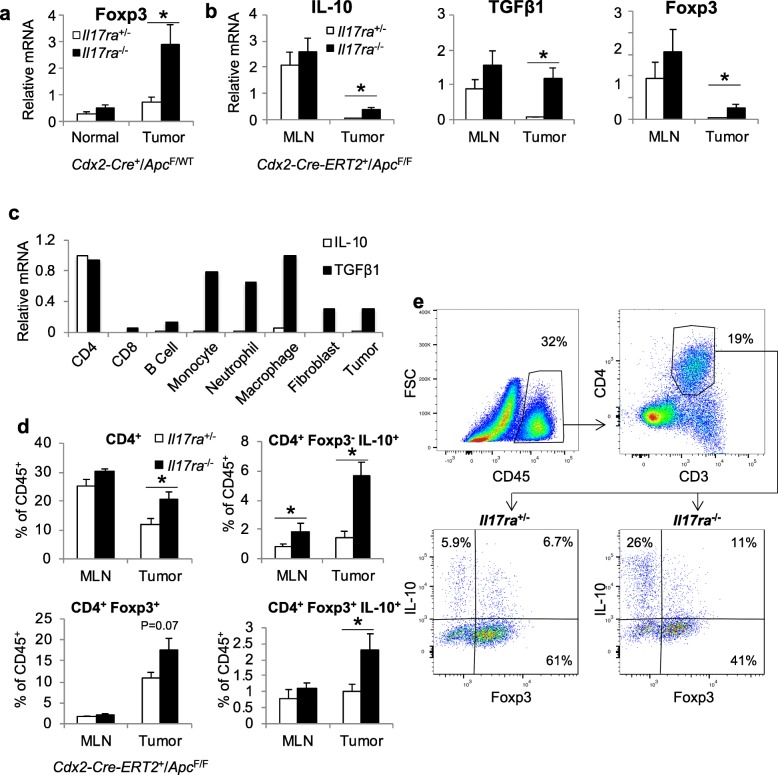
IL-17 inhibits the infiltration of CD4^+^ T cells, and the production of TGF-β and IL-10 in CRC. **a** and **b** q-RT-PCR analyses of the indicated mRNAs in designated tissues of control and IL-17RA-deficient *Cdx2-Cre*^*+*^*/Apc*^*F/+*^ mice (**a**, *n* = 11), and *Cdx2-Cre-ERT2*^*+*^*/Apc*^*F/F*^ mice (**b**, *n* = 5 for MLN, 11 for tumor). Mice in **b** received tamoxifen injection and were kept for 5 weeks for the development of early CRC tumors. Tumor-adjacent colonic tissues were used as “normal” control. **c** CD4^+^ T cells (CD45^+^ CD3^+^ CD4^+^), CD8^+^ T cells (CD45^+^ CD3^+^ CD8^+^), B cells (CD45^+^ CD19^+^), monocytes (CD45^+^ CD11b^+^ Ly-6C^High^), neutrophils (CD45^+^ CD11b^+^ Ly-6C^Low^, Ly-6G^+^), macrophages (CD45^+^ CD11b^+^, F4/80^+^), fibroblasts (CD45^−^ EpCam^−^), and tumor cells (CD45^−^ EpCam^+^) were FACS-sorted from pooled colonic tumors of 10 *Il17ra*^+/+^/*Cdx2-Cre*^*+*^*/Apc*^*F/+*^ mice. Purified cells were subjected to q-RT-PCR analysis, and the levels of each individual mRNA were shown as “1” in the cell type of highest expression. **d** *Cdx2-Cre-ERT2*^*+*^*/Apc*^*F/F*^ mice that were *Il17ra*^−/−^ or *Il17ra*^+/−^ were given i.p. injection of tamoxifen (75 mg/kg body weight) daily for 3 consecutive days. Mice were sacrificed 5 weeks after tamoxifen-induced *Apc* ablation, and their mesenteric lymph nodes (MLN) and tumors were subjected to flow cytometry analysis. *n* = 5. Cells were isolated following collagenase digestion of the indicated tissues, followed by 4-h in vitro stimulation with PMA and ionomycin in the presence of Brefeldin A and monensin. **e** Representative flow cytometry plots for tumors samples from **d**. Data represent means ± S.E.M. *p < 0.05 in Students’ *t* test

Using mouse models of sporadic and inducible CRC, we set out to understand how IL-17 regulates adaptive immunity. The mouse model of sporadic CRC is based on allelic inactivation of one copy of the *Apc* tumor suppressor gene in colonic epithelial cells that is driven by a *Cdx2-Cre* transgene (*Cdx2-Cre*^+^*/Apc*^F/+^) [24, 27]. Subsequent *Apc* loss-of-heterozygosity (LOH) results in the development of large colonic adenomas and adenocarcinomas in the gut [24]. Tumors in this model are restricted to the colon and rectum and progress to adenocarcinomas, which makes this model more relevant to human CRC than the commonly used *Apc*^MIN^ mice, where most of their tumors develop in the small intestine [28]. We also used a second model of synchronized colorectal tumorigenesis [25], which relies on tamoxifen-induced ablation of the *Apc* gene in *Cdx2-Cre-ERT2*^*+*^*/Apc*^F/F^ mice permitting the study of early stage colorectal tumorigenesis [25]. Early CRC lesions can be detected histologically 1 week after tamoxifen injection. If undisrupted, these early lesions progress to visible colorectal tumors by 4 weeks. Using these tools, we found that IL-17 inhibits the production of IL-10 and TGF-β, both of which limit Th17 activity and inhibit CRC development [1]. IL-10 and TGF-β are produced by multiple immune and stromal cells within tumors, including Tregs [29]. Ablation of IL-17RA in the sporadic CRC model resulted in an increased level of Foxp3, a key marker for Tregs (Fig. 1a). Since our previous study showed that IL-17 is critical for the growth of early CRC lesions, we also examined the levels of IL-10 and TGF-β1 in early CRC tumors that were only 1 to 2 mm in diameter. Ablation of IL-17 signaling resulted in profoundly (more than 10-fold) increased levels of IL-10 and TGF-β1 mRNAs (Fig. 1b), and significantly induced the expression of Foxp3 in the tumor (Fig. 1b), suggesting a major role of IL-17 signaling in the suppression of anti-inflammatory cytokine production during early stage CRC. TGF-β1 is produced by multiple cell types within the tumor microenvironment, whereas IL-10 production appears to be restricted to CD4^+^ T cells (Fig. 1c). In early CRC lesions, ablation of IL-17 signaling resulted in increased recruitment of CD4^+^ T cells to the tumors and elevated numbers of IL-10^+^ CD4^+^ T cells that are either Foxp3^+^ (Tregs) or Foxp3^−^ (Tr1 cells) (Fig. 1d, e). These data indicate that IL-17 inhibits the infiltration of Treg cells and the production of anti-inflammatory cytokines in early stage CRC.

### IL-17 inhibits the infiltration of CTLs in early stage CRC

**Fig. 2:**
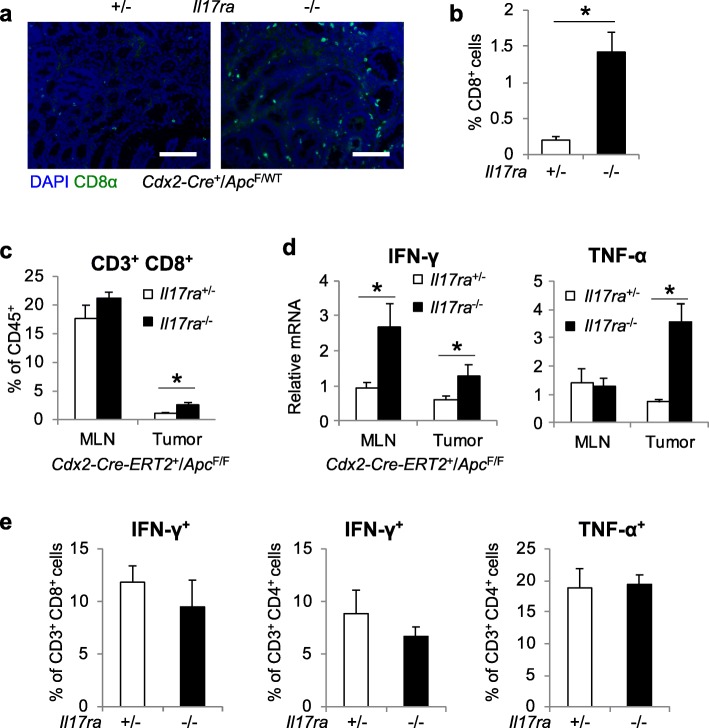
IL-17 blocks the accumulation of CD8^+^ T lymphocytes starting from early stage CRC. **a** Immunostaining of colon tumors from 5-month-old *Cdx2-Cre*^*+*^*/Apc*^*F/+*^ mice that were heterozygous (+/−) or null (−/−) for *Il17ra*. Scale bar = 100 μm. **b** Statistics for the percentages of CD8^+^ cells shown in **a**. *n* = 8. **c-e**: *Cdx2-Cre-ERT2*^*+*^*/Apc*^*F/F*^ mice that were *Il17ra*^+/−^ or *Il17ra*^−/−^ were sacrificed 5 weeks after tamoxifen-induced *Apc* ablation, and their MLN (**c** and **d**) and tumors (**c**, **d**, and **e**) were subjected to flow cytometry (**c** and **e**, *n* = 4 for *Il17ra*^+/−^, 10 for *Il17ra*^−/−^) and q-RT-PCR (**d**, *n* = 5 for MLN, 11 for tumor) analyses. Data represent means ± S.E.M. *p < 0.05 in Students’ *t* test

We have previously shown that IL-17 inhibits the expression of Th1/Tc1 signature genes [1]. This may result from IL-17-mediated inhibition on the infiltration of CD8^+^ CTLs to CRC. Indeed, immunostaining of cryosectioned colonic tumors showed that ablation of IL-17RA resulted in a marked increase in the number of CD8^+^ T cells in sporadic colorectal tumors (Fig. 2a, b). To test if this inhibition of CTL infiltration by IL-17 happens in early stage CRC, we performed flow cytometry analysis on tumors that developed following tamoxifen-induced deletion of *Apc* in colonic epithelium. Loss of IL-17RA in this model also resulted in marked elevation in the number of CD8^+^ CTLs in tumors (Fig. 2c), demonstrating an inhibitory role of IL-17 signaling in limiting CTL infiltration in early stage colon tumors. Early tumors that lost IL-17RA also exhibited elevated expression of IFN-γ and TNF-α (Fig. 2d). IL-17 signaling has no direct impact on the production of IFN-γ and TNF-α by T cells (Fig. 2e). Since CD8^+^ CTLs were long known to function in cancer immunesurveillance [30], the observed inhibition of CD8^+^ T cell infiltration by IL-17 is consistent with IL-17’s role in promoting early stage CRC development [1].

### IL-17 suppresses the expression of CXCL9, 10, and 11

**Fig. 3:**
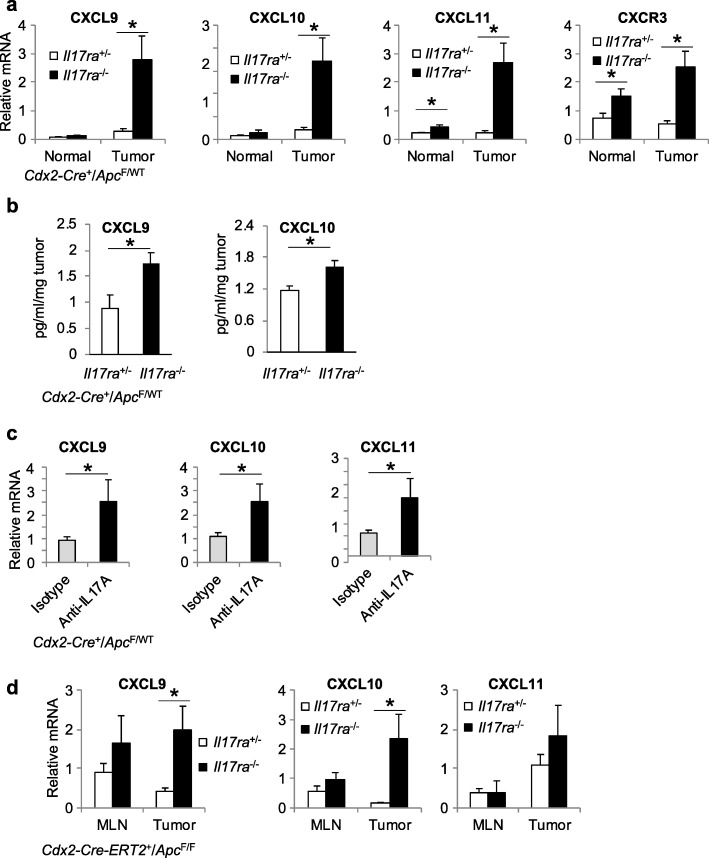
IL-17 inhibits the production of CXCL9 family chemokines. **a** q-RT-PCR analyses of the indicated mRNAs in normal colon and colorectal tumor tissues of 5-month-old control (*Il17ra*^*+/−*^) and IL-17RA whole body knockout (*Il17ra*^*−/−*^) mice that also harbor *Cdx2-Cre*^*+*^*/Apc*^*F/+*^ genotypes (*n* = 12). **b** Colonic tumors from control (*Il17ra*^*+/−*^) and IL-17RA-deficient (*Il17ra*^*−/−*^) *Cdx2-Cre*^*+*^*/Apc*^*F/+*^ mice were cultured in Opti-MEM medium for 24 h. Concentrations of chemokines were tested using a bead-based immunoassay (Biolegend, for CXCL9), or plate based ELISA (R&D systems, for CXCL10). Data are shown as pg/ml chemokine per mg tumor in culture (*n* = 13). **c** 5-month-old *Cdx2-Cre*^*+*^*/Apc*^*F/+*^ mice were treated with *i.p.* injection of isotype or anti-IL-17A antibodies (100 μg per injection, every 3 days) for two weeks. Colonic tumors were harvested at the end of the study, and analyzed by q-RT-PCR for indicated mRNAs. *n* = 13. **d** *Cdx2-Cre-ERT2*^*+*^*/Apc*^*F/F*^ mice (that were *Il17ra*^−/−^ or *Il17ra*^+/−^) were sacrificed 5 weeks after tamoxifen-induced *Apc* ablation. Mouse MLN and tumors were subjected to q-RT-PCR analysis (*n* = 5 for MLN, 11 for tumor). Data represent means ± S.E.M. *p < 0.05 in Students’ *t* test

IL-17 is known to promote chemokine production and attraction of MDSCs to tumors [1, 7, 31, 32]. Ablation of IL-17RA in mice resulted in reduced intratumoral levels of CXCL1 and 2 [1], consistent with the known role of IL-17 in promoting CXCL1/2 production and myeloid cell recruitment [33, 34]. Whether IL-17 regulates T cell recruitment is unknown. We found that loss of IL-17 signaling resulted in increased levels of the T cell-attracting chemokines CXCL9, 10, and 11 in colonic tumors (Fig. 3a, b). Loss of IL-17 signaling also increased the expression of CXCR3, the cognate receptor for CXCL9/10/11 chemokines, likely reflecting an increased recruitment of CXCR3-expressing lymphocytes to the tumor (Fig. 3a). Upregulation of the CXCL9 family of chemokines was also seen in 5-month-old CRC tumors of mice receiving *i.p.* injections of anti-IL-17A neutralizing antibody, demonstrating the effect of acute IL-17A blockade in chemokine production (Fig. 3c). Consistent with the notion that IL-17 inhibits T cell infiltration during early stage CRC, ablation of its receptor in the mouse model of induced colorectal tumorigenesis resulted in elevated CXCL9 family chemokines in early CRC tumors (Fig. 3d).

### IL-17 signals to transformed colonic epithelial cells to suppress the production of CXCL9, 10, and 11

**Fig. 4:**
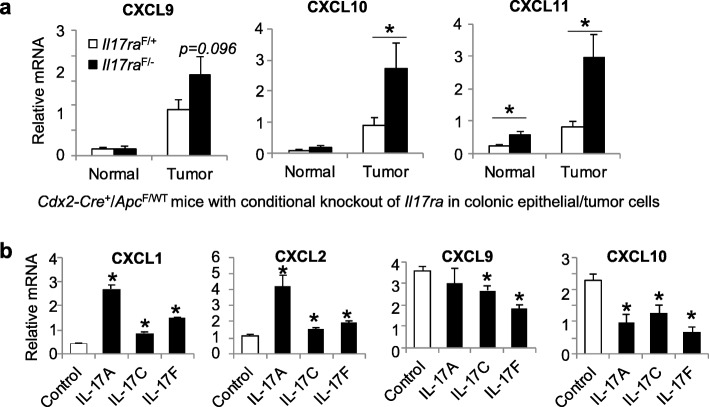
IL-17 signals to transformed epithelial cells to suppress CXCL9 family chemokine production. **a** *Cdx2-Cre*^*+*^*/Apc*^*F/+*^ mice were crossed to *Il17ra-flox* mice to generate a conditional ablation of *Il17ra* gene in colorectal epithelial cells and tumor cells. These mice carry *Cdx2-Cre*^*+*^*/Apc*^*F/+*^*/Il17ra*^F/−^ genotypes and are labeled as “*Il17ra*^F/−^”. *Cdx2-Cre*^*+*^*/Apc*^*F/+*^*/Il17ra*^F/+^ mice (labeled as *Il17ra*^F/+^) were used as controls. Both groups of mice were sacrificed at 5 months of age. Colorectal tumors and adjacent normal colon tissues were harvested for q-RT-PCR analysis. *n* = 6. **b** Tumor cells were isolated from colorectal tumors of *Cdx2-Cre-ERT2*^+^*/Apc*^F/F^ mice 4 weeks after tamoxifen injection. Cells were then cultured a 3-D system to allow their development into primary tumor spheres. Tumor spheres were subsequently treated with vehicle control (PBS with 0.1% BSA) or 100 ng/ml recombinant human IL-17A, C, or F for 24 h, followed by q-RT-PCR analysis (*n* = 3, and data represent one of three consistent tests). Data represent means ± S.E.M. *p < 0.05 in Students’ *t* test

We have previously shown that IL-17 mainly signals to transformed colonic epithelial cells (tumor cells) to promote CRC development [1]. It is possible that the same signaling pathway also controls CXCL9 family chemokine production. To this end, we analyzed the expression of CXCL9 family chemokines in *Cdx2-Cre*^*+*^/*Apc*^F/WT^ mice that harbor colon epithelial cell-specific deletion of IL-17RA. Loss of IL-17 signaling to colonic epithelial cells and their transformed counterparts resulted in elevated expression of CXCL9 family chemokines (Fig. 4a). To confirm a direct inhibitory effect of IL-17 on the production of chemokines, we isolated tumor cells from *Cdx2-Cre-ERT*^*+*^/*Apc*^F/F^ mice, and cultured these cells in Matrigel to form tumor spheres. These tumor spheres were treated with recombinant IL-17A, C or F, and then analyzed for chemokine expression by q-RT-PCR. Consistent with the previously known role of IL-17 in promoting the production of myeloid cell attracting chemokines, treatment with recombinant IL-17 resulted in elevated levels of CXCL1 and 2 (Fig. 4b). However, in primary tumor spheres IL-17 stimulation resulted in reduced levels of CXCL9 and 10 mRNAs (Fig. 4b), thus confirming a direct inhibitory role of IL-17 on CXCL9/10 production.

### CXCR3 signaling attracts CTLs and Treg cells to inhibit CRC development

**Fig. 5:**
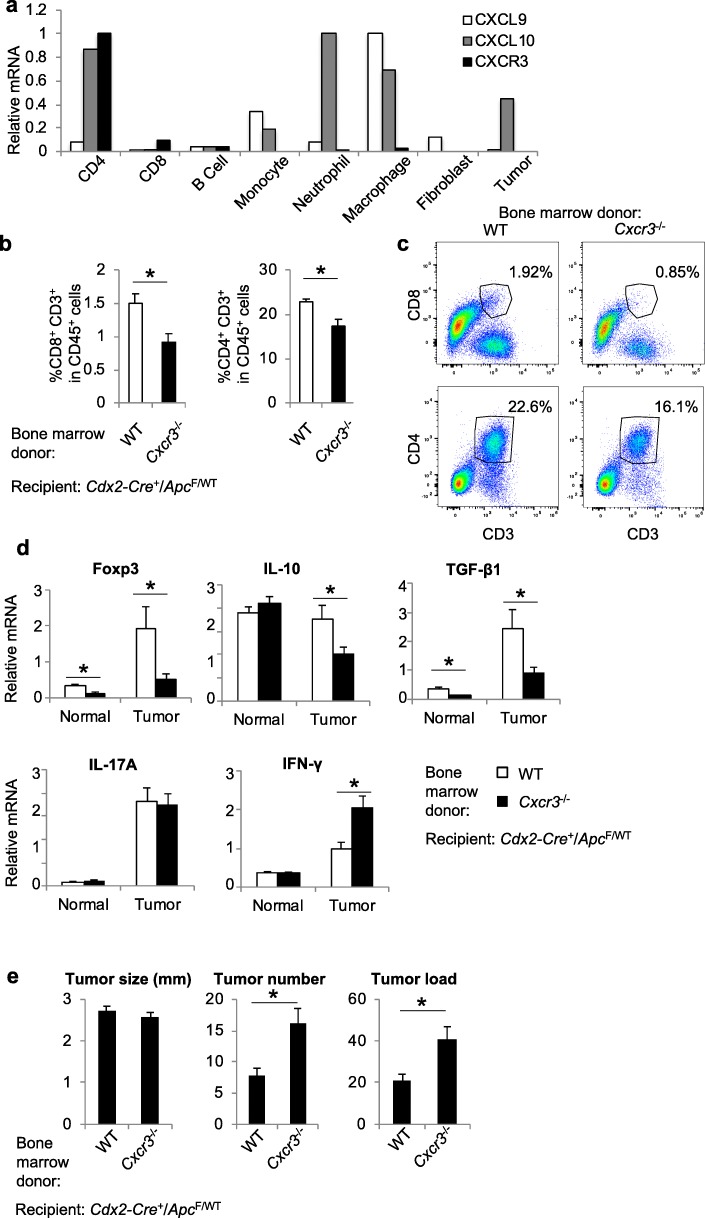
CXCR3 mediates the attraction of CD8^+^ CTLs and Treg cells, and inhibits the CRC development. **a** FACS-purified cells (as depicted Fig. 1c) from colonic tumors of *Cdx2-Cre*^*+*^*/Apc*^*F/*WT^ mice were subjected to q-RT-PCR analysis. **b**-**e** Bone marrow cells were harvested from WT and *Cxcr3*^−/−^ mice, and transferred into lethally irradiated 6–8-week-old *Cdx2-Cre*^+^/*Apc*^F/WT^ mice. Recipient mice were sacrificed at 5 months of age, and their colorectal tumors were used for flow cytometry (**b** and **c**, *n* = 7), q-RT-PCR (**d**, *n* = 16), and tumor statistics (**e**, *n* = 9). Cells shown in **c** were gated as live/CD45^+^. Data represent means ± S.E.M. *p < 0.05 in Students’ *t* test

CXCL9 and 10 are expressed by tumor cells and tumor-infiltrating myeloid cells, and their receptor CXCR3 is restricted to T lymphocytes (Fig. 5a). We reasoned that this chemokine pathway may be responsible for IL-17’s inhibitory role in T cell migration to CRC. Indeed, ablation of CXCR3 in all blood cells by means of bone marrow reconstitution resulted in reduced recruitment of CD8^+^ T cells, and to a lesser extent, Tregs to colorectal tumors (Fig. 5b, c). Ablation of CXCR3 in blood cells also reduced the levels of IL-10 and TGF-β in tumors (Fig. 5d), both of which have been shown to inhibit CRC development by dampening tumor-promoting inflammation [15, 17, 35, 36]. CXCR3 ablation also resulted in a marked decrease in the level of Foxp3 in tumors (Fig. 5d), suggesting a reduced Treg recruitment upon loss of CXCR3. CXCR3 signaling in hematopoietic cells is dispensable for the recruitment of Th1, Th17, and myeloid cells to colorectal tumors (Additional file 1: Figure S1). Ablation of CXCR3 in bone marrow cells showed no impact on IL-17 expression in tumors, and resulted in elevated IFN-γ levels (Fig. 5d). Loss of CXCR3 also did not impact the activation and expansion of tumor-infiltrating T cells (Additional file 1: Figure S2). Consistent with the known role of CD8^+^ CTLs in limiting cancer development, loss of these cells in mice that lack Cd8α or Beta-2-Microglobulin (a subunit of MHC I complex) both resulted in elevated tumor development in the large intestine (Additional file 1: Figure S3a, b). Given the role of CXCR3 in mediating CD8^+^ CTL and Treg recruitment, we reasoned that loss of CXCR3 should also result in accelerated colorectal tumorigenesis. Indeed, mice that lack CXCR3 in hematopoietic cells developed increased numbers of colorectal tumors, without changes in tumor sizes (Fig. 5e). The expression levels of CXCL9 family chemokines were not affected by the loss of CD8^+^ T cells in CRC-bearing mice (Additional file 1: Figure S3c), suggesting that these cells are not required for the production of CXCL9 family chemokines. Overall, these data show that CXCR3 signaling selectively attracts CD8^+^ CTLs and Tregs to CRC, and inhibits CRC development.

### IL-17 blockade upregulates the expression of immune checkpoint markers

**Fig. 6:**
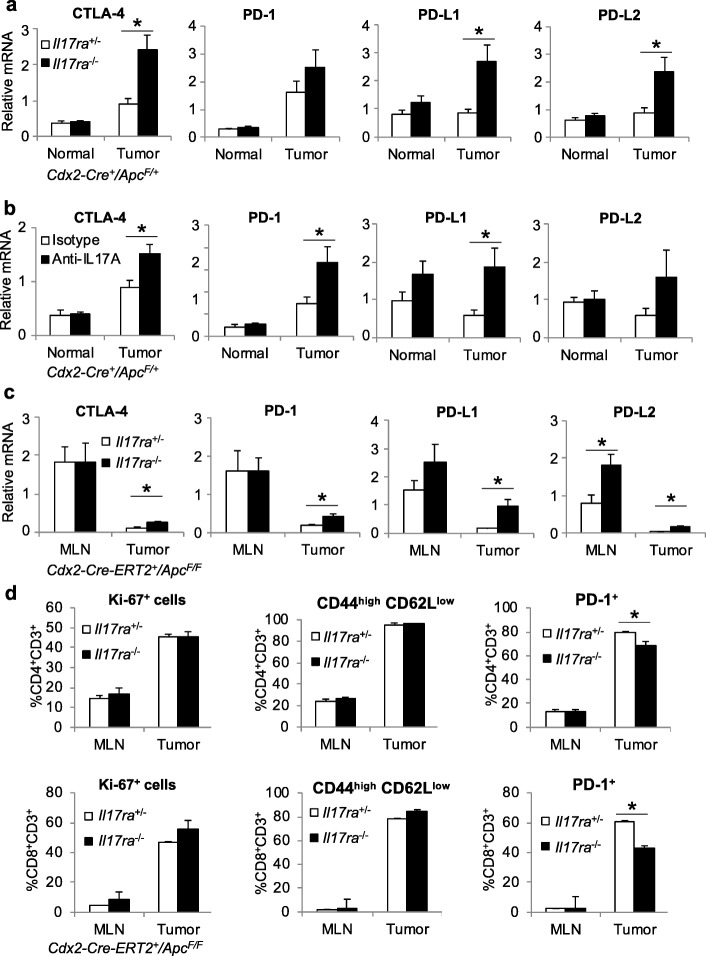
Blockade of IL-17 signaling in CRC results in enhanced immune checkpoint signaling. **a** q-RT-PCR analysis of normal colon tissue and colorectal tumors from *Cdx2-Cre*^*+*^*/Apc*^*F/+*^ mice that harbor heterozygous or null alleles of *Il17ra* gene (*n* = 12). **b** 4-month-old *Cdx2-Cre*^*+*^*/Apc*^*F/+*^ mice received i.p. injection of 100 μg isotype or anti-IL-17A antibodies every 3 days for 1 month. Mice were sacrificed for q-RT-PCR analysis (*n* = 9). **c** *Cdx2-Cre-ERT2*^*+*^*/Apc*^*F/F*^ mice that were *Il17ra*^+/−^ or *Il17ra*^−/−^ were sacrificed 5 weeks after tamoxifen-induced *Apc* ablation, and their MLN and tumors were subjected to q-RT-PCR analysis (*n* = 5 for MLN, 11 for tumor). **d** *Cdx2-Cre-ERT2*^*+*^*/Apc*^*F/F*^ mice that were *Il17ra*^−/−^ or *Il17ra*^+/−^ were sacrificed 5 weeks after tamoxifen-induced *Apc* ablation, and their MLN and tumors were subjected to flow cytometry analysis. *n* = 5. Data represent means ± S.E.M. *p < 0.05 in Students’ *t* test

**Fig. 7:**
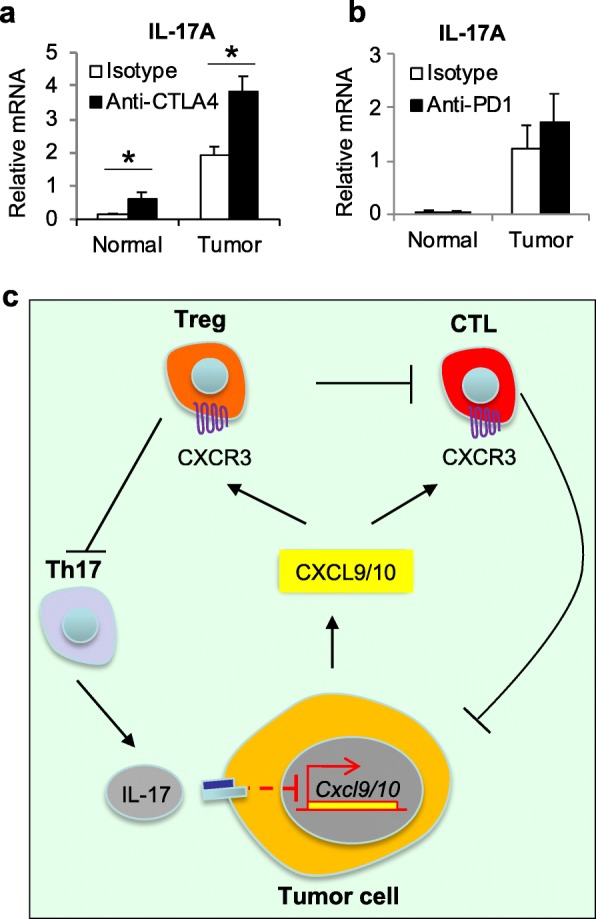
Blockade of CTLA-4 induced the expression of IL-17 in CRC. **a**, **b** 5-month-old *Cdx2-Cre*^*+*^*/Apc*^*F/+*^ mice received *i.p.* injection of 100 μg isotype or blocking antibodies against CTLA-4 (**a**, *n* = 24) or PD-1 (**b**, *n* = 8) every 3 days for 2 weeks, and were sacrificed for q-RT-PCR analysis. Data represent means ± S.E.M. *p < 0.05 in Students’ *t* test. **c**: IL-17 signals directly to tumor cells in CRC to inhibit the production of CXCL9 and CXCL10. These two chemokines are required for the recruitment of CD8^+^ CTLs and Tregs, which inhibits CRC development by targeting cancer cells and dampening tumor-promoting inflammation, respectively

Immune checkpoint inhibitors, such as antibodies that block CTLA-4 and PD-1 signaling, are effective only in a fraction of colorectal cancers that show microsatellite instability (MSI) [37, 38]. Given the role of IL-17 in inhibiting the infiltration of CTLs and Treg cells to CRC, we next tested if its blockade would impact immune checkpoint signaling. Ablation of IL-17RA in the mouse sporadic tumor model resulted in elevated expression of CTLA-4 (Fig. 6a), a cell surface protein that is constitutively expressed in Tregs and mediates part of their immune suppressive function [39, 40]. In addition, IL-17RA-null tumors also exhibited elevated expression levels of PD-L1 and PD-L2 (Fig. 6a). Similar changes were observed in *Cdx2-Cre*^+^/*Apc*^F/WT^ mice treated with neutralizing antibody against IL-17A (Fig. 6b). Upregulation of CTLA-4 and PD-1 pathway markers were also observed in the mouse model of early stage colorectal tumorigenesis (Fig. 6c), suggesting an antagonism of IL-17 and immune checkpoint pathways beginning at the early phase of CRC development. We have previously shown that ablation of IL-17RA in CRC led to an increased level of IFN-γ [1], which is known to upregulate PD-L1 expression [41]. IL-17 signaling does not impact proliferation or activation of tumor-infiltrating CD4^+^ and CD8^+^ T cells (Fig. 6d). Both tumor-infiltrating CD8^+^ and CD4^+^ T cells express PD-1, and the proportion of PD-1-positive T cells decreased modestly upon ablation of IL-17 signaling (Fig. 6d). Therefore, the increase in overall PD-1 expression in CRC likely reflects the substantial increase in T cell infiltration upon blockade of IL-17 signaling, and not increased PD-1 expression on a per cell basis. CTLA-4 immunotherapy has been tested in human cancers and showed variable efficacy [42, 43]. Consistent with the role of Tregs in limiting tumor-associated inflammation, blockade of CTLA-4 signaling by antibody increased the expression of IL-17A in tumors (Fig. 7a). In contrast, expression of IL-17 was not changed in mice that received blocking antibody for PD-1 (Fig. 7b). These results suggest that CTLA-4 blockade upregulates pro-tumor IL-17 in colorectal tumors. Taken together, our data show that IL-17 signals to tumor cells to downregulate the production of chemokines CXCL9/10, which are required for attracting CD8^+^ CTLs and Tregs to CRC. Inhibition of CXCL9/10 signaling by IL-17 thus reduces the activity of anti-cancer immunity, and fosters stronger tumor-promoting inflammation (Fig. 7c).

## Discussion

IL-17 is known to promote neutrophil infiltration by activating the production of their attracting chemokines. In mouse model of CRC, ablation of IL-17 resulted in reduced levels of CXCL1 and CXCL2, which correlates with decreased numbers of tumor infiltrating myeloid cells [1, 4, 5, 44]. We also showed that these tumor-infiltrating myeloid cells respond to bacterial products that pass through defective surface barrier due to tumorigenesis, and produce IL-23 [3]. IL-23 in turn promotes the production of IL-17 by T cells and innate lymphoid cells [3]. In this way, IL-17 and tumor-infiltrating myeloid cells form an auto-enhancing loop to promote tumor-associated inflammation. Combined with our new finding that IL-17 inhibits T cell infiltration through the downregulation of CXCL9/10, it is now clear that IL-17 skews tumor immune environment towards an innate cell-dominant, tumor-promoting inflammation. In different settings, IL-17 has also been shown to promote the infiltration and develop of myeloid-derived suppressor cells (MDSC), which inhibit the activity of CTLs and thus promotes tumor development [31, 45]. The contribution of MDSC to T cell inactivation in sporadic CRC is unknown, but may represent an alternative pathway by which IL-17 indirectly inhibits CD8^+^ CTL activity. It is therefore possible that tumor-infiltrating myeloid cells play dual roles in CRC: 1) these cells respond to commensal bacteria and promote tumor-associated inflammation (such as the production of IL-23 and IL-17), which subsequently leads to reduced CXCL9/10 production and T cell attraction; 2) these cells may serve as suppressors of anti-tumor immunity. Additional research is required to dissect the inflammation-promoting v.s. T cell inactivating roles of myeloid cells in tumors. For example, one may employ myeloid-specific ablation of effector molecules (such as arginase [45]) to examine the effect of MDSC in sporadic CRC.

Chemokine signaling through CXCR3 has been shown to inhibit tumor growth in several transplantable tumor models [10, 11, 46]. This anti-tumor function of CXCR3 and its cognate ligands were attributed to the recruitment of CD8^+^ CTLs into tumors. Consistently, in human CRC, a high CXCL10 level correlates with CD8^+^ T cell infiltration [47]. In our study, we also observed reduced CTL number in colorectal tumors upon ablation of CXCR3 in hematopoietic cells. In contrast, CXCR3 signaling was dispensable for Th1 and Th17 cell infiltration. Intriguingly, we found that CXCR3 functions to recruit Treg cells to CRC tumors, and CXCR3 loss results in marked decreases in the levels of IL-10 and TGF-β. Given the anti-tumor roles of IL-10 and TGF-β in early stage colon cancer development, we concluded that CXCR3 inhibits early stage colorectal tumorigenesis by attracting both CTLs and Treg cells. This conclusion was supported by the observation that loss of CXCR3 in blood cells resulted in increased tumor incidence in mouse colon, but no changes in tumor size. It is also in agreement with the known role of IL-17 in promoting early stage CRC development [1].

In this study, we report a novel mechanism by which IL-17 inhibits the recruitment of CD8^+^ CTLs and Treg cells by downregulating the production of CXCL9/10 chemokines. Such knowledge will demonstrate the feasibility of interfering with IL-17-Treg interaction for CRC prevention and immunotherapy. For instance, blockade of IL-17 signaling may be useful for the prevention of CRC in genetically susceptible populations, such as FAP (familial adenomatous polyposis) patients that harbor germline mutations in the *Apc* tumor suppressor gene. Given the known role of IL-17 in promoting early stage CRC development [1], and its negative impact in the inhibition of CD8^+^ CTLs and Tregs, blocking IL-17 may suppress tumor-promoting inflammation, activate tumor immunosurveillance, and reduce the rate of tumorigenesis in this genetically predisposed population.

Immunotherapy against human CRC has shown limited success, as it is effective only in microsatellite instable (MSI) cases [37, 38]. For the 85% of microsatellite stable CRC, checkpoint inhibition largely does not work. Our mouse models of CRC are based on allelic inactivation of the *Apc* tumor suppressor gene [24, 25, 27], and do not carry MSI lesions. Yet, in both sporadic and early stage CRC models, ablation of IL-17 signaling resulted in increased recruitment of anti-tumor CD8^+^ CTLs via upregulation of CXCL9 family chemokines, without the requirement of MSI. It is possible that in human CRC that are microsatellite stable, blockade of IL-17 can also result in increased production of CXCL9 family chemokines and enhanced infiltration of CD8^+^ T cells to tumors, which is a desirable trait for cancer immunotherapy. Upregulation of IL-17 in mouse models of CRC stems from the loss of surface barrier function in the process of epithelium transformation. In this regard, it remains to be tested if IL-17 plays a similar role in limiting T cell infiltration in MSI tumors.

While IL-17 blockade may also increase the number of Tregs in human CRC, blockade of immune checkpoints should be sufficient to neutralize their inhibition on anti-cancer immunity. In this regard, neutralizing antibodies against IL-17A and IL-17RA, which have been tested safe and effective for the treatment of autoimmunity in humans [48], may be tested as adjuvant therapies that accompany current cancer immunotherapies. IL-17 production is restricted to the CRC tumor site, and its blockade should result in selective upregulation of CXCL9 family chemokines in tumors. In this perspective, IL-17 blockade should be effective in attracting T cells to tumors, and poses less risk of systemic immune activation.

## Conclusions

Our data show a novel role of IL-17 in inhibiting the infiltration of CD8^+^ CTLs and Tregs to CRC. This is mediated by IL-17’s signaling to colorectal tumor cells, which leads to the reduced production of CXCL9/10 chemokines. CXCL9/10 chemokines, signaling through their cognate receptor CXCR3, recruit CD8^+^ CTLs and Tregs to CRC, but are dispensable for the recruitment or activation of other T cells and myeloid cells. By excluding Tregs and CTLs from CRC, IL-17 fosters the dominance of tumor-promoting inflammation. To this end, cancer immunotherapy may be benefited by the use of anti-IL-17 agents, as blockade of IL-17 reduces the rate of tumor growth and increases the infiltration of CTLs that are vital for effective cancer treatment.

## Supplementary information

**Additional file 1: Table S1.** Primer Sequences for q-RT-PCR. **Figure S1.** CXCR3 signaling is dispensable for the recruitment of Th1, Th17 and myeloid cells. **Figure S2.** CXCR3 signaling is dispensable for the activation of T cells. **Figure S3.** CD8^+^ T cells inhibit the development of sporadic CRC.

## Data Availability

All data generated or analyzed during this study are included in this published article and its supplementary information files.

## Abbreviations

B2m: Beta-2-microglobulin
CRC: Colorectal cancer
CTL: Cytotoxic T lymphocytes
CTLA4: Cytotoxic T-lymphocyte-associated protein 4
CXCL10: C-X-C Motif Chemokine Ligand 10
CXCL11: C-X-C Motif Chemokine Ligand 11
CXCL9: C-X-C Motif Chemokine Ligand 9
Foxp3: Forkhead box P3
IFN-γ: Interferon gamma
IL-10: Interleukin-10
IL-17: Interleukin-17
IL-17A: Interleukin-17A
IL-17C: Interleukin-17C
IL-17F: Interleukin-17F
IL-17RA: Interleukin-17 receptor A
MDSC: Myeloid-derived suppressor cells
MLN: Mesenteric lymph node
PD-1: Programmed cell death-1
PD-L1: Programmed death-ligand 1
PD-L2: Programmed death-ligand 2
Th1: Type 1 T helper cells
Th17: T helper 17 cells
TNF-a: Tumor Necrosis Factor-α
Treg: Regulatory T cells
