# Supplementary material for: IL-17 inhibits CXCL9/10-mediated recruitment of CD8+ cytotoxic T cells and regulatory T cells to colorectal tumors
Source: J Immunother Cancer. 2019 Nov 27;7:324. doi: 10.1186/s40425-019-0757-z (PMC6880503; doi:10.1186/s40425-019-0757-z)
Supplement: Supplementary file 1 — Additional file 1: Table S1. Primer Sequences for q-RT-PCR. Figure S1. CXCR3 signaling is dispensable for the recruitment of Th1, Th17 and myeloid cells. Figure S2. CXCR3 signaling is dispensable for the activation of T cells. Figure S3. CD8+ T cells inhibit the development of sporadic CRC. [file 40425_2019_757_MOESM1_ESM.pdf]

Table S1: Primer Sequences for q-RT-PCR		
mRNA	Reverse primer	Forward primer
RPL32	TTGTGAGCAATCTCAGCACA	GGGAGCAACAAGAAAACCAA
IL-10	ACCTGCTCCACTGCCTTGCT	GGTTGCCAAGCCTTATCGGA
TGF-β1	AAGTTGGCATGGTAGCCCTT	GGAGAGCCCTGGATACCAAC
Foxp3	TCCAAGTCTCGTCTGAAGGC	GCGAAAGTGGCAGAGAGGTA
IFN-γ	GCGTCATTGAATCACACCTG	GAGCTCATTGAATGCTTGGC
TNF-α	GGTCTGGGCCATAGAACTGA	CAGCCTCTTCTCATTCCTGC
CXCL9	AGTCCGGATCTAGGCAGGTT	GAGGCACGATCCACTACAAA
CXCL10	CCTATGGCCCTCATTCTCAC	CGTCATTTTCTGCCTCATCC
CXCL11	CATTTTGACGGCTTTCATCC	AAGGTCACAGCCATAGCCCT
CXCR3	AAGTGCCAAAGGCAGAGAAG	CAAAGTCCGAGGCATCTAGC
CXCL1	TCTCCGTTACTTGGGGACAC	CCACACTCAAGAATGGTCGC
CXCL2	CTTTGGTTCTTCCGTTGAGG	CAAAAAGTTTGCCTTGACCC
CTLA-4	CTGAAGGTTGGGTCACCTGT	TGGACTCCGGAGGTACAAAG
IL-17A	GCCCTCAGACTACCTCAACC	ACACCCACCAGCATCTTCTC
PD-1	CCTCTGACACTGTGAGCCAG	GCAGGTACCCTGGTCATTCA
PD-L1	ATGCTCAGAAGTGGCTGGAT	TGCTGCATAATCAGCTACGG
PD-L2	CAAGCCTCAGCCTAGCAGAA	CCGGGATGAAAACATGAAGT

Figure S1

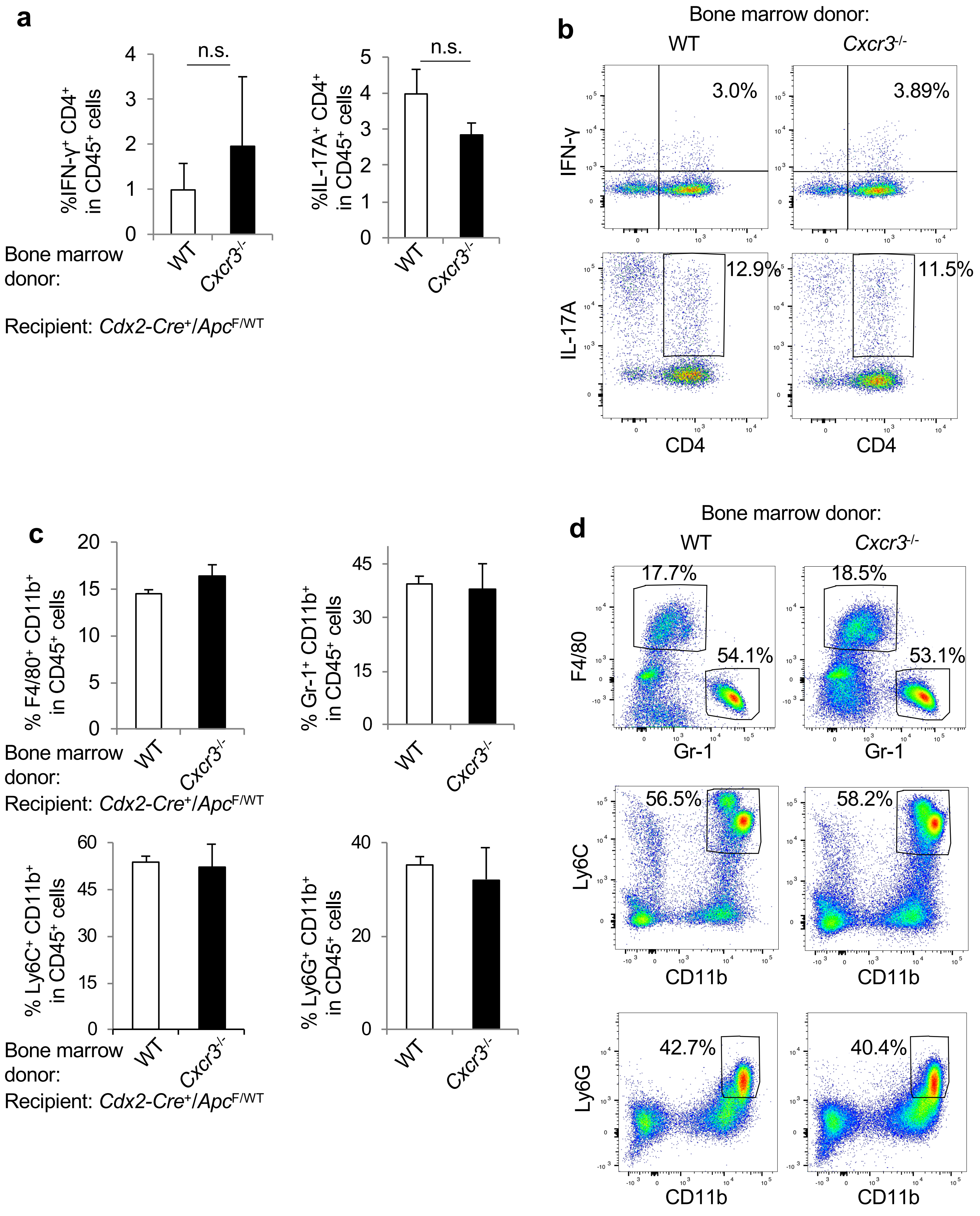

a
n.s.
%IFN-γ+ CD4+ in CD45+ cells
%IL-17A+ CD4+ in CD45+ cells
Bone marrow donor:
WT
Cxcr3-/-
Recipient: Cdx2-Cre+/ApcF/WT
b
Bone marrow donor:
WT
Cxcr3-/-
3.0%
3.89%
12.9%
11.5%
IFN-γ
IL-17A
CD4
c
% F4/80+ CD11b+ in CD45+ cells
% Gr-1+ CD11b+ in CD45+ cells
% Ly6C+ CD11b+ in CD45+ cells
% Ly6G+ CD11b+ in CD45+ cells
Bone marrow donor:
WT
Cxcr3-/-
Recipient: Cdx2-Cre+/ApcF/WT
d
Bone marrow donor:
WT
Cxcr3-/-
17.7%
54.1%
18.5%
53.1%
F4/80
Gr-1
56.5%
58.2%
Ly6C
CD11b
42.7%
40.4%
Ly6G
CD11b

Figure S1: CXCR3 signaling is dispensable for the recruitment of Th1, Th17 and myeloid cells.

Bone marrow cells were harvested from WT and *Cxcr3*$^{-/-}$ mice, and transferred into lethally irradiated 6-8-week-old *Cdx2-Cre*$^{+}$/*Apc*$^{F/WT}$ mice. Recipient mice were sacrificed at 5 months of age, and their tumors were used for flow cytometry analyses. **a**: Percentage of IFN-γ or IL-17A positive CD4^{+} T cells in total CD45^{+} cells in colonic tumors. n=7. **b**: Representative plots of flow cytometry analysis in **a**. Cells were gated as live/CD45^{+}/CD3^{+}. **c**: Percentage of F4/80, Gr-1, Ly6C, or Ly6G positive cells that were CD11b^{+} in tumor-infiltrating CD45^{+} cells. n=6 for WT, 5 for *Cxcr3* knockout. **d**: Representative flow cytometry plots of **c**. Plots of F4/80 and Gr-1 staining (upper panel of **d**) were gated on CD45^{+} CD11b^{+} cells; plots of Ly6C and Ly6G staining (middle and lower panels of **d**) were gated on CD45^{+} cells. Data represent means ± S.E.M.

Figure S2

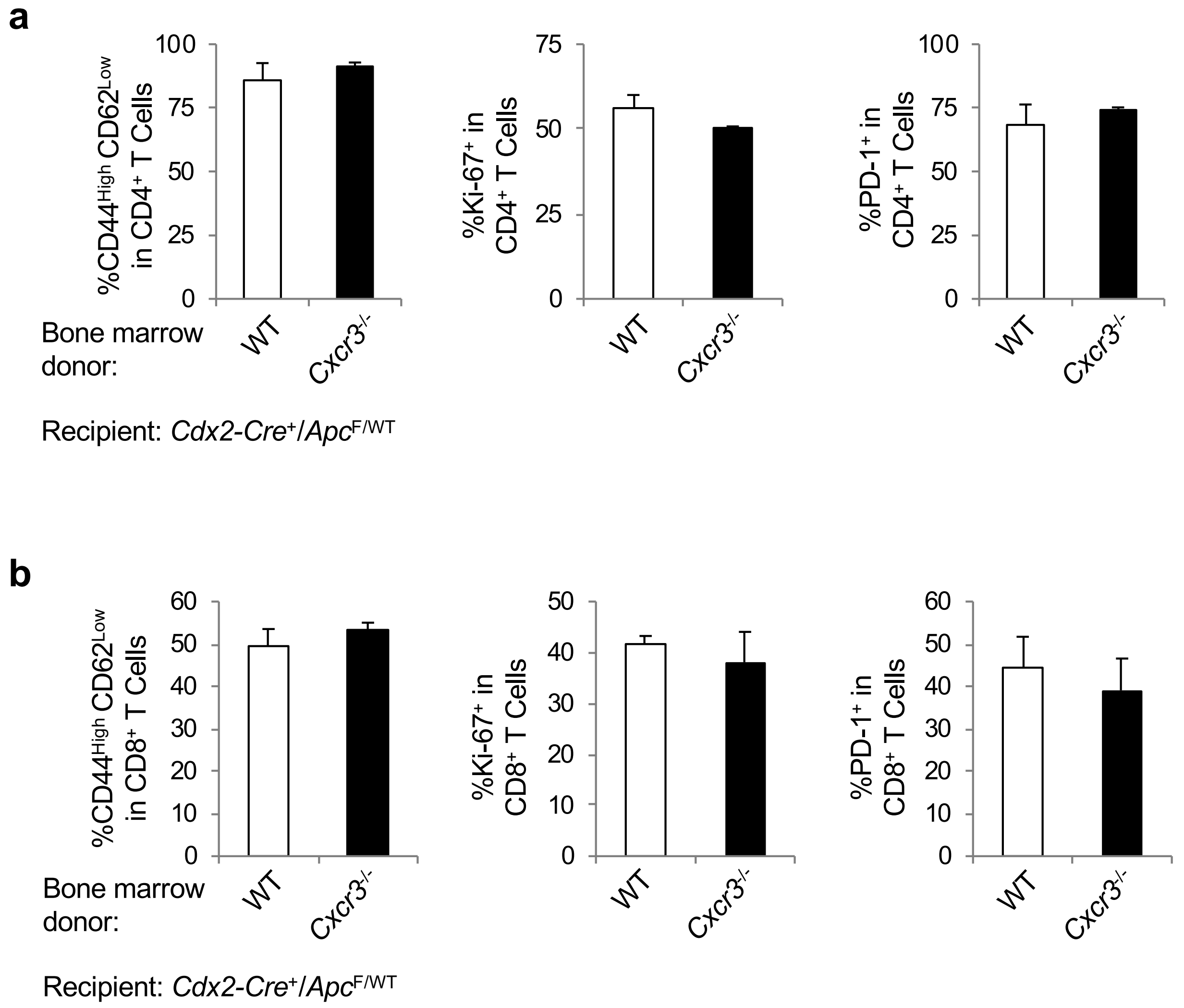

Figure S2: CXCR3 signaling is dispensable for the activation of T cells.
Bone marrow cells were harvested from WT and *Cxcr3*$^{-/-}$ mice, and transferred into lethally irradiated 6-8-week-old *Cdx2-Cre*$^{+}$/*Apc*$^{F/WT}$ mice. Recipient mice were sacrificed at 5 months of age, and their tumors were used for flow cytometry analyses. Dissociated tumor cells were stained and gated on live/CD45^{+} cells, and the percentages of indicated markers among CD4^{+} (**a**) or CD8^{+} (**b**) T cells are shown. n=5. Data represent means ± S.E.M.

Figure S3

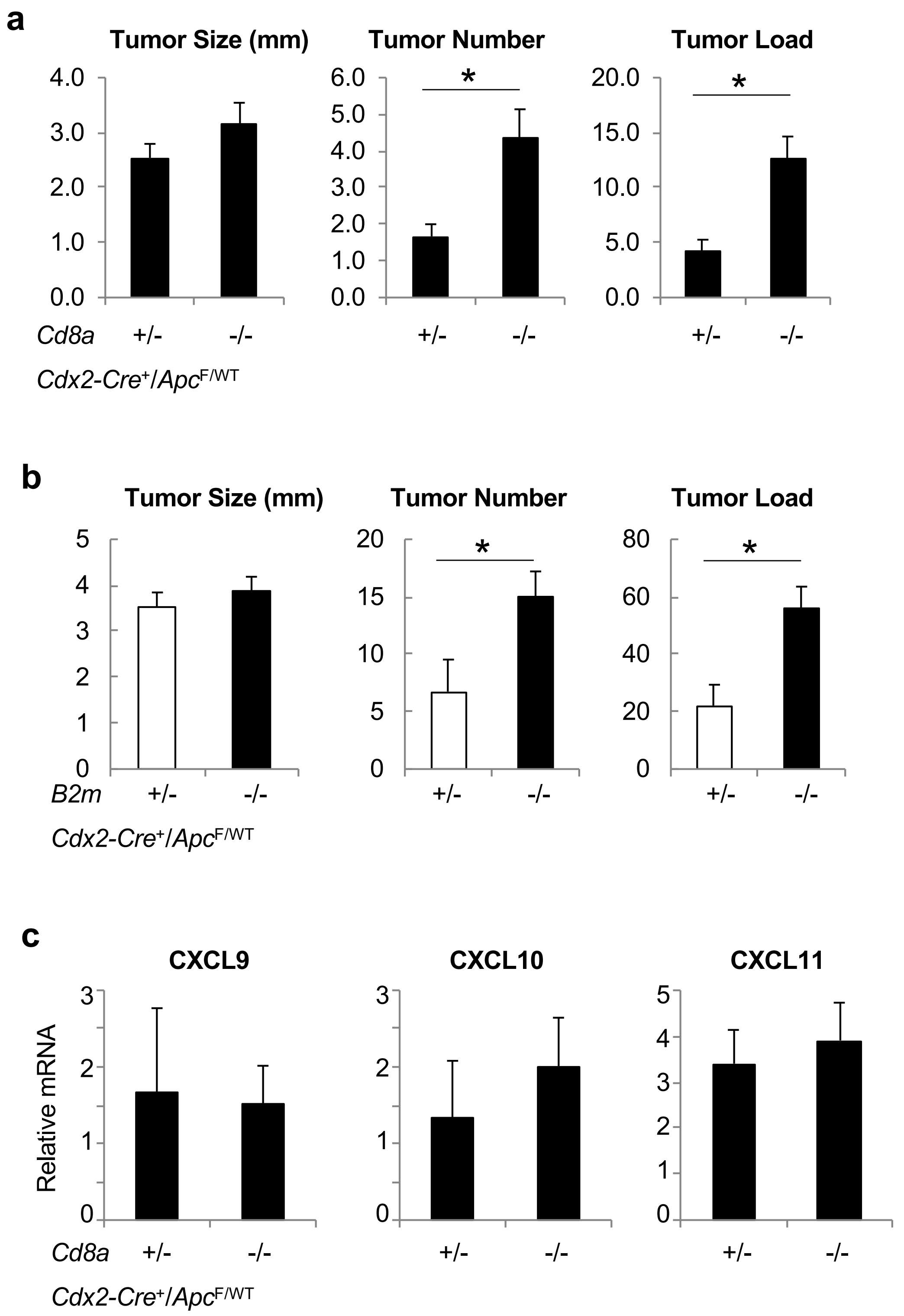

Figure S3: CD8^{+} T cells inhibit the development of sporadic CRC.
Cd8a$^{-/-}$ and *B2m*$^{-/-}$ mice were crossed to *Cdx2-Cre* and *Apc*$^{F/F}$ strains to generate mice that carry knockout of CD8 and Beta-2-microglobulin (B2m, a component of MHC I) in the mouse model of sporadic CRC. Mice were sacrificed at 5 months of age for colorectal tumor statistics (**a**, n=6 and **b**, n=7) and q-RT-PCR analysis on tumor samples (**c**). Data represent means ± S.E.M. *p<0.05 in Students' *t* test.
